# Psychological Distress, Anxiety, Depression, and Associated Factors Among Nigerian Healthcare Workers During COVID-19 Pandemic: A Cross-Sectional Study

**DOI:** 10.3389/ijph.2022.1604835

**Published:** 2022-11-17

**Authors:** Olanrewaju Ibikunle Ibigbami, Adesanmi Akinsulore, Tolu Opakunle, Champion Seun-Fadipe, Olakunle Ayokunmi Oginni, Victor Ogbonnaya Okorie, Ibidunni Oloniniyi, Olushola Olibamoyo, Olutayo Olubunmi Aloba, Boladale Mapayi, Abiodun Adewuya

**Affiliations:** ^1^ Department of Mental Health, College of Health Sciences, Obafemi Awolowo University, Ile-Ife, Nigeria; ^2^ Department of Mental Health, Obafemi Awolowo University Teaching Hospitals Complex, Ile-Ife, Nigeria; ^3^ State Specialist Hospital, Asubiaro, Oshogbo, Nigeria; ^4^ Leicestershire Partnership NHS Trust, Leicester, United Kingdom; ^5^ Department of Agricultural Extension and Rural Development, Obafemi Awolowo University, Ile-Ife, Nigeria; ^6^ Department of Behavioural Medicine, Lagos State University College of Medicine, Lagos, Nigeria

**Keywords:** psychological distress, nurses, doctors, COVID 19 pandemic, anxiety, depression, Nigeria

## Abstract

**Objectives:** We determined the prevalence of psychological distress, and the associations between sociodemographic factors, anxiety, depression, COVID-19-related experiences, and psychological distress, among nurses and doctors in Nigeria.

**Methods:** The study was a cross-sectional descriptive study, conducted over a month (1st of July–31st of July 2021) among 434 Health Care Workers (HCWs) [225 (51.8%) nurses and 209 (48.2%) doctors] from two tertiary health facilities in southwestern Nigeria. Binary logistic regression was carried out to determine the factors associated with psychological distress (dependent variable), while the independent variables were anxiety, depression, and COVID-19 experience-related factors.

**Results:** The prevalence of moderate and severe psychological distress was 49.1% and 5.8%, respectively. Individuals who had the first degree had significantly lower odds (AOR: 0.43; *p* = 0.037) of experiencing psychological distress while being a nurse (AOR: 2.03; *p* = 0.014), higher levels of anxiety (1.28; *p* < 0.001), and depression (AOR: 1.17; *p* = 0.005) were associated with significantly higher odds of experiencing moderate to severe levels of psychological distress.

**Conclusion:** There is a high level of psychological distress experienced by these health workers. Hence, they will benefit from strategies to reduce their distress.

## Introduction

The coronavirus disease 2019 (COVID-19) was declared a pandemic by the World Health Organization in March 2020. As of June 2022, more than 500 million people have been infected, with over 6.3 million deaths worldwide [1]. The pandemic has not only affected the physical health of individuals, but it has also had a tremendous impact on the psychosocial health of different populations and groups [2]. Specifically, it has disrupted healthcare services and caused substantial morbidity among healthcare personnel [3]. The COVID-19 pandemic has made healthcare workers (HCWs) more prone to psychosocial distress and other mental disorders [4]. This is because HCWs are besieged by psychosocial distress, fatigue, and occupational burnout [5].

In studies reported from different parts of the world including Africa, the prevalence of psychological distress among HCWs ranged from 27.3% to 80.7% [4, 6]. The figures are also expectedly high among Nigerian HCWs with rates ranging from 23.4% to 64.9% [7, 8].

The current COVID-19 pandemic has also been linked to many psychosocial problems including anxiety, depression, insomnia, exacerbations of mental illness, social isolation, feelings of helplessness, and neglect among HCWs [9] which are each associated with psychological distress. Specifically, the prevalence rates of anxiety and depression among HCWs during the COVID-19 pandemic ranged from 9.5 to 73.3%, and 12.5–71.9%, respectively [10]. More so, the measures put in place to combat the pandemic have disrupted social connection and deterioration in the overall social capital and connectedness among HCWs [11].

Some of the work-related factors that are associated with psychological distress among HCWs include shortage of personal protective equipment, longer working hours, overwhelming workloads, inadequate organizational support, and a rising number of cases [12]. Other general pandemic-related factors include concerns about transmitting the infection to family members, exposure to the disease, widespread media coverage of the pandemic, social discrimination, social isolation, and vaccine hesitancy [13]. Given these challenges, HCWs face complicated decisions and work under extreme pressures that might also evoke ethical issues, such as situations of triage, lack of experience in treating critical illness, inadequate palliative care, and inability to support relatives of terminally ill patients adequately [14]. These challenges and conditions might amplify HCWs’ psychological distress [15].

There is still a paucity of data on psychological distress among HCWs in low-resource settings such as Nigeria. Therefore, this study aimed to access self-reported psychological distress and associated factors among a sample of Nigerian HCWs. Our specific objectives were 1) to determine the prevalence of psychological distress among the study subjects; 2) to determine the associations between psychological distress and sociodemographic factors, mental health status (anxiety and depression), COVID-19-related work experiences, and 3) identify factors independently associated with psychological distress among the study population. Our goal was to explore the theoretical framework proposed by Pearlin et al. [16] which posits that stress can arise from discrete events and the presence of continuous problems. Individual HCW’s unique work experiences (discrete events) during this pandemic could have predisposed them to experience psychological distress, while the ongoing pandemic and its related life events (continuous problem) may have triggered significant psychological distress which may warrant requisite coping responses and targeted psychological intervention. We, therefore, hypothesized that there will be significant associations between COVID-19 related work experience, mental health status, and psychological distress among this sample of HCWs. Our findings will inform policymakers on the burden of psychosocial distress among Nigerian HCWs and may perhaps serve as an initial step at providing interventions to reduce this burden among them.

## Methods

### Study Design and Participants

The study adopted a cross-sectional descriptive study design, conducted *via* self-administered questionnaires for the quantitative baseline data of a research initiative towards the development of a mobile-health intervention for reducing psychological distress among HCWs in Nigeria. The project (GECO Destress NG project) is a bi-centre study conducted in two tertiary hospitals in Southwestern Nigeria: Obafemi Awolowo University Teaching Hospital Complex (OAUTHC), Ile-Ife, and Lagos State University Teaching Hospital (LASUTH), Lagos. OAUTHC is in a semi-urban town of Ile-Ife, while LASUTH is in the metropolitan city of Lagos, Nigeria’s commercial capital. The questionnaires were administered over the period of a month (1st of July–31st of July).

The sample size for the study was estimated using the Kish’s formula [17]: *n* = z^2^p (1 − p) ÷ d^2^; where n = required sample size, z = confidence level at 95% (standard value of 1.96), p = estimated prevalence rate was 38.5% (proportion of healthcare workers with psychiatric morbidity) [18], and d = margin of error at 5% (standard value of 0.05). We, therefore, estimated a minimum sample size of 364 respondents, and this was increased to 437 (20% added to make up for possible incomplete data).

A stratified random sampling method employing probability proportionate to size was used to recruit the study participants from each study site. In each hospital, only participants belonging to the medical and nursing professions were considered eligible to be included in the study. The OAUTHC is a Federal Government-owned tertiary hospital with 465 doctors and 887 nurses, while LASUTH is a State Government-owned tertiary hospital with 536 doctors and 987 nurses. The number of participants per profession from each study site was determined by proportional sampling to ensure equal representation from all the subgroups. Potential participants were randomly selected from the pool of eligible respondents across the two groups of health professionals from the nominal role by a table of random numbers, until the sample size is reached. Participants with severe mental or physical illness were excluded from the study, as participation may increase physical discomfort for those with severe physical illness while those with severe mental illness may not be able to give informed consent. These were clarified by asking directly from the respondents during the preliminary interactions with the respondents.

### Measurement

#### Sociodemographic and Self-Reported COVID-19 Related Experiences Questionnaire

The first section of the study instrument collected information on sociodemographic variables from the study participants which include age, sex (male, female), marital status (single, married, previously married), religion (Christianity, Islam, others), qualification (diploma, first degree, master’s degree, fellowship) medical history (good health, poor health) history of COVID infection and others. We also inquired about COVID-19 related experiences. Specific questions include “have you ever tested positive to COVID-19?” (response: Yes or No); “Have you had contact(s) with COVID-19 patients?” (responses: never, rarely, sometimes, always); “do you think that you have a history of exposure to the COVID-19 virus?” (responses: Yes or No); “Have any of your patients ever tested positive?” (No, I don’t know, yes); and “Have you ever thought of resigning?” (response: yes or no).

#### Kessler Psychological Distress Scale (K6):

The K6 is a 6-item instrument designed by Kessler et al. [19] to measure psychological distress. Each item relates to an emotional state or experiences during the past 30 days, and it is rated on a 5 point Likert scale from “none of the time (0)” to “all of the time (4)”, yielding a minimum score of 0 and a maximum score of 24. Scores of 0–4 indicated none-to-low distress, values of 5–12 indicated moderate distress, and values of ≥13 indicated severe psychological distress [20]. This scale has been proven to have cross-cultural reliability and validity [21]. For the binary logistic regression, the scores were dichotomized into “0” (none-low psychological distress) and “1” (moderate to severe psychological distress). The Cronbach’s alpha for the scale in this population was 0.73.

#### The Patient Health Questionnaire (PHQ-9)

This is a 9-item self-rated tool widely used for screening, diagnosing, monitoring, and measuring the severity of depression, which is based on the nine criteria in the depression module of DSM-IV [22]. Each item explores depressive symptoms over the preceding two weeks, and each is rated from “0” (not at all) to “3” (nearly every day) based on severity, with a minimum global score of 0 and a maximum score of 27. PHQ-9 scores of 5, 10, 15, and 20 represented mild, moderate, moderately severe, and severe depression, respectively [22, 23]. The Cronbach’s alpha for the scale in the present sample was 0.83.

#### Generalized Anxiety Disorder Questionnaire (GAD-7)

This is a 7-item self-report scale developed by Spitzer et al. [24] used for screening, and severity assessment of anxiety disorder [24]. Items are rated on a 4-point Likert-type scale (0 = not at all to 3 = nearly every day), with scores ranging from 0 to 21 with higher scores indicating more severe GAD symptoms. It has been reported that scores of 5, 10, and 15 are the cut-off points for mild, moderate, and severe anxiety, respectively [23]. The Cronbach alpha for the scale in the study sample was 0.86.

### Statistical Analysis

Data were analyzed using the IBM-SPSS version 25 software for Windows. The data were summarized using descriptive statistics such as frequency distributions, proportions, and mean. Chi-square (χ2) was used to compare the differences between dichotomous variables, while analysis of variance was used to compare mean scores across the categories of psychological distress. For the binary logistic regression model, the outcome (dependent) variable was psychological distress, while the explanatory (independent) variables were anxiety and depressive symptoms and work/COVID-19 experience related factors while sociodemographic variables were the confounders. Statistical significance was set at *p* < 0.05 in all cases.

### Ethical Consideration

Ethical approval for the research was obtained from the Ethics and Research Committees of OAUTHC and LASUTH. Furthermore, the study protocol was reviewed with a favorable ethical opinion by the Liverpool School of Tropical Medicine Research Ethics Committee. The researchers sought informed consent from participants, and confidentiality was maintained, as data was obtained and stored anonymously.

## Results

A total of 434 health professionals participated in this study. Their ages ranged between 22 and 64 years with a mean (SD) age of 37.4 (9.08) years. The subjects from the two study sites were; OAUTHC- 226 (52.1%) and LASUTH- 208 (47.9%). Across the professions, there were slightly more nurses (225 = 51.8%), than doctors (209 = 48.2%). A greater number of the sample were “younger than 40 years” (274; 63.1%), female (291; 67.1%), married (322; 74.2%), and of the Christian faith (372; 85.7%). Most of them had a first degree (317; 73.0%) as their highest qualification, and self-reported having “good health” (397; 91.5%) according to their medical history. The prevalence of moderate and severe psychological distress was 49.1% and 5.8%, respectively, while the prevalence of “moderate to severe” anxiety and depression symptoms were 3.2% and 6.7%, respectively. A significantly greater proportion of the females (χ^2^ = 11.82, *p* = 0.003), nurses (χ^2^ = 20.33, *p* < 0.001) and respondents with first degree (χ^2^ = 17.92, *p* = 0.006) had experienced psychological distress compared to those who were male, doctors and those having lower academic qualifications respectively. Also, individuals who had “moderate to severe” levels of psychological distress had significantly higher levels of symptoms of anxiety (χ^2^ = 49.47, *p* < 0.001) and depression (χ^2^ = 52.50, *p* < 0.001). These are presented in Table 1.

**TABLE 1:** Associations between sociodemographic factors, mental health status and psychological distress among health workers; total number (*N*) = 434 (Ile-Ife and Ikeja, Nigeria. 2021).

Variable	Total	Psychological distress			Statistics
	N (%)/Mean (SD)	None	Moderate	Severe	*x*2 (p value)/F test (p value)
		*N* = 196 (45.2%)	*N* = 213 (49.1%)	*N*= 25 (5.8%)	
Age (in years)					
<40	274 (63.1)	127 (64.8)	131 (61.5)	16 (64.0)	0.48 (0.785)
≥40	160 (36.9)	69 (35.2)	82 (38.5)	9 (36.0)	
Sex					
Male	143 (32.9)	81 (41.3)	57 (26.8)	5 (20.0)	**11.82 (0.003)**
Female	291 (67.1)	115 (58.7)	156 (73.2)	20 (80.0)	
Religion					
Christianity	372 (85.7)	165 (84.2)	186 (87.3)	21 (84.0)	1.65 (0.800)
Islam	59 (13.8)	30 (15.3)	25 (11.7)	4 (16.0)	
Others	3 (0.7)	1 (0.5)	2 (0.9)	0 (0.0)	
Marital Status					
Single	104 (24.0)	50 (25.5)	49 (23.0)	5 (20.0)	1.25 (0.869)
Married	322 (74.2)	143 (73.0)	160 (75.1)	19 (76.0)	
Previously married (Widowed/Divorced/Separated)	8 (1.8)	3 (1.5)	4 (1.9)	1 (4.0)	
Hospital Site					
OAUTHC	226 (52.1)	104 (53.1)	113 (53.1)	9 (36.0)	2.75 (0.253)
LASUTH	208 (47.9)	92 (46.9)	100 (46.9)	16 (64.0)	
Profession					
Doctor	209 (48.2)	117 (59.7)	85 (39.9)	7 (28.0)	**20.33 (**< **0.001)**
Nurse	225 (51.8)	79 (40.3)	128 (60.1)	18 (72.0)	
Qualification					
Diploma	49 (11.3)	11 (5.6)	32 (15.0)	6 (24.0)	**17.92 (0.006)**
First Degree	317 (73.0)	152 (77.6)	148 (69.5)	17 (68.0)	
Master’s degree	35 (8.1)	13 (6.6)	21 (9.9)	1 (4.0)	
Fellowship	33 (7.6)	20 (10.2)	12 (5.6)	1 (4.0)	
Medical History					
Good Health	397 (91.5)	183 (46.1)	193 (90.6)	21 (84.0)	2.90 (0.235)
Poor Health	37 (8.5)	13 (6.6)	20 (9.4)	4 (16.0)	
Anxiety	2.30 (3.20)	0.92 (1.73)	3.17 (3.53)	5.72 (3.97)	**49.47 (<0.001)**
Depression	2.96 (3.71)	1.35 (2.03)	3.93 (4.08)	7.24 (4.40)	**52.50 (<0.001)**

Bold values denote statistically significant values at *p*-value <0.05.

With regards to COVID-19 related factors, a total of 57 (13.1%) study participants had tested positive to COVID-19, while more than half (54.4%) admitted to having had contacts with COVID-19 positive patients “sometimes” (48.1%) or “always” (6.3%). More than 6 out of every 10 of the participants (297, 68.6%) thought that they had a history of exposure. A total of 294 (67.7%) of the study participants had had patients who tested positive for COVID-19 infection, while the majority of them (286, 65.9%) were worried that they will be infected. Participants who had experienced being avoided by family and friends, and thoughts of resigning were 125 (28.8%) and 48 (11.1%), respectively.

Most of the study participants (254, 58.5%) had received formal training on COVID-19. Those who were aware that their hospitals had a COVID-19 safety protocol, a triage protocol, and a response team were 395 (91.0%), 355 (81.8%), and 401 (92.4%), respectively. A significantly greater number of those who had; tested positive to COVID-19 (χ^2^ = 7.52, *p* = 0.027), admitted to having had a history of exposure (χ^2^ = 10.26 *p* = 0.006), had patients who had tested positive for COVID-19 (χ^2^ = 13.22, *p* = 0.010), had been worried about being infected (χ^2^ = 13.73, *p* = 0.001), had family and friends who avoided them (χ^2^ = 13.03, *p* = 0.001), and had experienced thoughts of resigning (χ^2^ = 33.97, *p* < 0.001) were found to have experienced moderate to severe levels psychological distress compared to those who did not have these experiences. These are presented in Table 2 below.

**TABLE 2:** Association between COVID-19 related factors, mental health status and psychological distress among health workers; total number (*N*) = 434 (Ile-Ife and Ikeja, Nigeria. 2021).

Variable	Total	Psychological distress			Statistics
	N (%)/Mean (SD)	None	Moderate	Severe	x2 (p value)/F test (p value)
		N= 196 (45.2%)	N= 213 (49.1%)	N= 25 (5.8%)	
Ever tested positive for COVID-19					
No	377 (86.9)	177 (90.3)	182 (85.4)	18 (72.0)	**7.52 (0.027)**
Yes	57 (13.1)	19 (9.7)	31 (14.6)	7 (28.0)	
Contacts with confirmed COVID-19 patients					
Never/Rarely	140 (32.3)	72 (36.7)	61 (28.6)	7 (28.0)	3.28 (0.194)
Sometimes/Always	294 (67.7)	124 (63.3)	152 (71.4)	18 (72.0)	
Previous exposure to COVID-19					
No	136 (31.4)	75 (38.3)	58 (27.4)	3 (12.0)	**10.26 (0.006)**
Yes	297 (68.6)	121 (61.7)	154 (72.6)	22 (88.0)	
Any of your patients ever tested positive					
I don’t know	53 (12.2)	31 (15.8)	16 (7.5)	6 (24.0)	**13.22 (0.010)**
No	87 (20.0)	44 (22.4)	41 (19.2)	2 (8.0)	
Yes	294 (67.7)	121 (61.7)	156 (53.1)	17 (68.0)	
Being worried that you will be infected					
No	148 (34.1)	83 (42.3)	62 (29.1)	3 (12.0)	**13.73 (0.001)**
Yes	286 (65.9)	113 (57.7)	151 (70.9)	22 (88.0)	
Family and friends avoided you					
No	309 (71.2)	155 (79.1)	141 (66.2)	13 (52.0)	**13.03 (0.001)**
Yes	125 (28.8)	41 (20.9)	72 (33.8)	12 (48.0)	
Ever thought of resigning?					
No	386 (88.9)	188 (95.9)	183 (85.9)	15 (60.0)	**33.97 (<0.001)**
Yes	48 (11.1)	8 (4.1)	30 (14.1)	10 (40.0)	

Bold values denote statistically significant values at *p*-value <0.05.

As shown in Table 3, having a first degree (AOR: 0.43; *p* = 0.037) was associated with significantly lower odds of experiencing psychological distress. The factors associated with greater odds of experiencing moderate to severe levels of psychological distress include being a nurse (AOR: 2.03; *p* = 0.014), higher levels of anxiety (1.28; *p* < 0.001), and depression (AOR: 1.17; *p* = 0.005).

**TABLE 3:** Binary logistic regression analysis to determine the association between psychological distress, sociodemographic variables, and COVID-19 related experiences among health workers in Nigeria; total number (*N*) = 434 (Ile-Ife and Ikeja, Nigeria. 2021).

Variables	Psychological distress (reference- No distress) AOR (95% CI)	*p*-value
Sex		
Female (Ref- Male)	1.26 (0.71–2.20)	0.430
Profession		
Nurse (Ref- Doctor)	2.03 (1.16–3.55)	**0.014**
Qualification		
Diploma	Reference	
First Degree	0.43 (0.19–0.95)	**0.037**
Master’s	0.77 (0.26–2.28)	0.632
Fellowship	0.38 (0.12–1.26)	0.114
Anxiety	1.28 (1.12–1.46)	**<0.001**
Depression	1.17 (1.05–1.30)	**0.005**
Ever tested positive to COVID-19		
Yes (Ref: No)	1.81 (0.89–3.70)	0.104
History of exposure to COVID-19		
Yes (Ref: No)	1.40 (0.82–2.37)	0.219
Patient tested positive to COVID-19		
Yes (Ref: No)	1.44 (0.87–2.39)	0.157
Worried about getting infected with COVID-19		
Yes (Ref: No)	1.13 (0.69–1.86)	0.625
Thought of resigning		
Yes (Ref: No)	2.17 (0.86–5.46)	0.102
Families and friends avoiding contact		
Yes (Ref: No)	1.39 (0.82–2.38)	0.220
Cox & Snell R2	0.273s	
Nagelkerke R2	0.365	
Omnibus test of model coefficients	137.58	**<0.001**
Hosmer lemeshow test	13.73	0.089

Bold values denote statistically significant values at *p*-value <0.05.

## Discussion

The present study found that 54.9% of healthcare workers who participated in the present study had moderate to high levels of psychological distress and this was significantly higher among those who were female, practiced as nurses, did not have postgraduate qualifications, and had high levels of depressive and anxiety symptoms. COVID-19- and work-related factors associated with psychological distress included testing positive for COVID-19 infection, self-reported exposure to COVID-19 positive patients, “treating COVID-19 positive patients,” “worrying about getting infected with COVID-19,” “being avoided by family and friends,” and “considering the possibility of resigning.” Of these, practicing as a nurse, lower academic qualifications, and experiencing higher depressive or anxiety symptoms were independently associated with psychological distress. These findings fully support our hypothesis.

The high rate of psychological distress found among healthcare workers in the present study is consistent with that found in Nigeria [7], in other low-and-middle-income countries [25] and high-income countries [26]. Our findings that the female subjects experience significantly higher levels of psychological distress is in keeping with previous studies that have explored gender differences in the general population [27], and the distress associated with the untoward experiences in response to the ongoing COVID-19 pandemic [28]. This has been reported among the general population and among populations of health workers in developed and developing countries. Women tend to experience more psychological distress because of role strain, work balance problems, lack of sufficient support systems, and unique challenges of pregnancy and motherhood [29]. While men could experience more disruptions than women in some aspects of their life [30], the overall experience of psychological distress has consistently been more among the women [28]. This may also reflect the higher vulnerability of women for internalizing problems [31] while men may be more likely to manifest distress with externalizing problems [32].

We also showed that being a nurse increased the likelihood of experiencing higher levels of psychological distress. This finding is similar to what has been reported by previous researchers [33], with nursing being described as an occupational factor in the experience of psychological distress during the pandemic [34]. The work experience of nurses in these settings might be more unfavorable compared with that of doctors. Generally, in Nigeria, HCWs are grossly underpaid, overworked, and being made to work under extreme conditions of inadequate infrastructure and motivation, making their work experience to be untoward. This has triggered an increase in the number of these professionals who are migrating out of the country to greener pastures [35]. Particularly, nurses have felt this impact more with studies showing their dissatisfaction with their work conditions. Despite the ongoing pandemic, the number of these highly skilled professions that are emigrating to Europe and America from Nigeria has continued to rise.

Higher academic qualifications may indicate a better capacity to cope with stress either from having undergone rigorous academic activities [36] or the consequent higher socio-economic status which might result in more educated individuals having better financial resources to cope with the stress of the pandemic. For example, at the beginning of the pandemic, healthcare staff had to individually purchase their personal protective equipment (PPE) when these are not adequately provided by the government [37]. Having just a diploma suggests that the health worker is a junior staff. Junior staff earn lesser income and must continue to function optimally despite the risks associated with the pandemic. This lesser pay and more work could have translated to a greater experience of psychological distress.

Our findings that the experience of anxiety and depression (which could both be psychological responses to untoward life events) were associated with higher levels of psychological distress among these HCWs is similar to findings from previous studies [38]. One possibility is that psychological distress and depressive and anxiety symptoms are jointly influenced by an underlying vulnerability [31]. Depressive and anxiety symptoms may also be later manifestations of severe psychological distress [39], although in this case, the strength of the associations between psychological distress and depressive/anxiety symptoms would be expected to be higher than those found in the present study. When individuals experience stressful life events (like this pandemic), the experience triggers off a cascade of coping responses which help to ameliorate the impact of the experience and forestall a breakdown of the individual’s mental health. However, when the coping mechanisms are overwhelmed, these could result in internalizing symptoms like anxiety or depression. These symptoms are expressed in the form of psychological distress [40]. Among HCWs, there is significant work-related stress. The ongoing pandemic has added to this stressful experience. In LAMICs like Nigeria, the pandemic-related stress factors include shortages of personal protective equipment, fear of getting infected, poor remuneration, and non-payment of allowances [41]. All these could have negatively impacted the morale of these professionals resulting in their experience of significant psychological distress.

Testing positive to the virus was also found to be significantly associated with psychological distress. This is in keeping with previous studies conducted among the general population and populations of frontline workers like HCWs [42]. These individuals are at increased risk of being infected due to work-related exposures. Currently, there is no cure for the virus worldwide. There is also a significant risk of mortality and long-term physical morbidities which do not have a clear definition of their extent. Hence, individuals who test positive for the virus, especially HCWs who would have more knowledge about the nature of the virus are likely to experience more psychological distress. Similarly, those who have had a history of exposure or having contact with patients who have tested positive for the virus will also experience psychological distress.

We also found out that persons who were worried about being infected had significantly higher levels of psychological distress. There is an association between worry and anxiety. Anxiety is fearful apprehension or appraisal of the potential of danger or threat to oneself. Individuals who are worried about a situation are more likely to experience psychological distress about that situation.

Consistent with prior research, specific COVID-19-related factors such as worry about infection, actual exposure to persons with COVID-19, and testing positive for COVID-19 were associated with higher psychological distress [43]. These associations have also been linked to worry about infecting family members, medical complications of infection, and possibly fatal outcomes [44]. This was more so considering repeated reports of healthcare providers dying from COVID-19-related morbidity [45] possibly acquired in the context of providing healthcare. The latter observation is consistent with our finding that treating patients with COVID-19 was significantly associated with higher psychological distress. A further correlate of psychological distress among healthcare workers in our study was stigma in the broader social context. Stigma has been previously demonstrated as a risk factor for psychological distress [46], however, our study suggests that COVID-19-related stigma is a further risk indicator for psychological distress and other adverse mental health outcomes among healthcare workers in Nigeria. A possible explanation for the higher rate of psychological distress among nurses in the present study compared to doctors is the longer contact time they have with patients especially for nurses who practice in in-patient rather than out-patient contexts [47], although this was not specifically assessed in the present study.

Having a first degree was found to be associated with low psychological distress compared to having a diploma. Within this population, individuals who have a first degree are more likely going to be either doctors or senior nurses. Their status puts them at a higher socioeconomic pedestal, with access to more finance, and other resources to meet their needs. This is likely to reduce their experience of psychological distress because access to finance and other resources has been found to reduce the stress associated with traumatic life events like the pandemic.

Our study has also shown that being a nurse increased the odds of experiencing psychological distress. This is in keeping with other studies [48]. The practice of nursing entails that the staff nurse spends more time in contact with the patients on admission [40]. This puts them more at risk of being infected. We could not find any significant association between gender and psychological distress. This shows that it is not the gender (most nurses are females), but the profession that might explain the experience of psychological distress. Our study has shown that both anxiety and depression are associated with psychological distress. This is in keeping with findings among populations of HCWs in other climes and the general population [40]. Globally, the world has witnessed at least four major waves of an upsurge in the rate of infection. More variants of the initial virus have also been identified. Despite the disbursement of vaccines, there had been a need to continue the already prescribed preventive measures for reducing the spread of the virus. With no end in sight to the pandemic, HCWs who are front liners in the fight against the virus are more likely to experience subjective feelings of apprehension, helplessness, and eventual burnout [49]. High levels of anxiety could reduce performance, while feelings of depression could be accompanied by disinterest and a general lack of the drive to perform optimally. All these will reduce the overall output of the health workers and a reduction in their performance which will induce work-related distress, keeping them in a vicious cycle of poor mental health and underproductivity.

The strength of our study lies in the fact that this is the first study multicenter study in Nigeria to explore psychological distress among health workers. The sample size is also robust enough to justify our findings. We also used standardized instruments to measure psychological distress and other variables that were assessed in this study. We acknowledge some limitations. Firstly, the study adopted a cross-sectional descriptive design which makes it difficult for us to infer a direct causal relationship between psychological distress and the other attributes measured. Secondly, the study participants were also asked to recount their past experiences during the pandemic. Individual bias or inexact recall might influence their accounts. Thirdly, we also limited our research to only doctors and nurses; other health professionals were not included. We limited our research to this population because these are the leading clinical staff, who directly attend to patients. We also did not explore differences in the subspecialties of the respondents, or their duty post differences, for instance, in-patient/out-patient post for nurses. However, these do not negate our findings as the process for obtaining the informed consent was detailed and allowed only participants who were willing to volunteer information to freely participate in the research.

### Conclusion

There is a high level of psychological distress experienced by these HCWs during the ongoing pandemic. The high level of psychological distress is associated with being a nurse and experiencing symptoms of anxiety and depression. This shows that this population of HCWs will benefit from strategies to improve their wellbeing and their professional competence. This justifies the need for the development of an innovative, culturally sensitive, cost-effective, and evidence-based treatment model for the psychological distress experienced by these HCWs.
